# Complex formation of EphB1/Nck/Caskin1 leads to tyrosine phosphorylation and structural changes of the Caskin1 SH3 domain

**DOI:** 10.1186/1478-811X-10-36

**Published:** 2012-11-27

**Authors:** Szabolcs Pesti, Annamária Balázs, Roopesh Udupa, Beáta Szabó, Anna Fekete, Gábor Bőgel, László Buday

**Affiliations:** 1 From the Department of Medical Chemistry, Semmelweis University Medical School, Budapest, 1094, Hungary; 2 Institute of Enzymology, Research Center for Natural Sciences, Hungarian Academy of Sciences, Budapest, 1113, Hungary

**Keywords:** EphB1 receptor tyrosine kinase, Nck, Caskin1, SH3 domain, CD spectrum

## Abstract

**Background:**

Scaffold proteins have an important role in the regulation of signal propagation. These proteins do not possess any enzymatic activity but can contribute to the formation of multiprotein complexes. Although scaffold proteins are present in all cell types, the nervous system contains them in the largest amount. Caskin proteins are typically present in neuronal cells, particularly, in the synapses. However, the signaling mechanisms by which Caskin proteins are regulated are largely unknown.

**Results:**

Here we demonstrate that EphB1 receptor tyrosine kinase can recruit Caskin1 through the adaptor protein Nck. Upon activation of the receptor kinase, the SH2 domain of Nck binds to one of its tyrosine residues, while Nck SH3 domains interact with the proline-rich domain of Caskin1. Complex formation of the receptor, adaptor and scaffold proteins results in the tyrosine phosphorylation of Caskin1 on its SH3 domain. The phosphorylation sites were identified by mass-spectrometry as tyrosines 296 and 336. To reveal the structural consequence of this phosphorylation, CD spectroscopy was performed. This measurement suggests that upon tyrosine phosphorylation the structure of the Caskin1 SH3 domain changes significantly.

**Conclusion:**

Taken together, we propose that the scaffold protein Caskin1 can form a complex with the EphB1 tyrosine kinase via the Nck protein as a linker. Complex formation results in tyrosine phosphorylation of the Caskin1 SH3 domain. Although we were not able to identify any physiological partner of the SH3 domain so far, we could demonstrate that phosphorylation on conserved tyrosine residues results in marked changes in the structure of the SH3 domain.

## Background

The Eph family is the largest family of receptor tyrosine kinases, at least, 14 different Eph kinases have been identified so far [1,2]. These receptors can be divided into two subclasses (EphB and EphA) based on the cell surface ligand which they interact with. Ligands that interact with EphA receptors are generally attached to the cell surface via glycosylphosphatidylinositol (ephrinA ligands), while ligands that activate EphB receptors are transmembrane proteins (ephrinB ligands) [1,2]. Eph receptor kinases and their ligands (ephrins) play a critical role in embryonic patterning, angiogenesis and neuronal targeting [2-4]. In the nervous system, EphB receptor kinases are enriched at excitatory synapses and are important during synapse and spine formation and maintenance [2].

Adaptor proteins, composed almost entirely of well-defined interaction domains, serve to link two functional members of a signaling pathway [5]. Nck family of adaptors comprising of SH2 and SH3 domains are implicated in the organization of actin cytoskeleton, cell movement, and axon guidance. Their SH2 domains bind specific phosphotyrosine residues on activated receptors or their substrates, whereas the SH3 domains bind proline-rich motifs on downstream target proteins [6]. It has been shown earlier that Nck associates with a number of Eph tyrosine kinases, including EphB1, EphB2, EphA2, EphA3 and EphA4 [7-12]. For EphB1, the first tyrosine residue localized in the juxtamembrane region represents the binding site for the SH2 domain of Nck, while its SH3 domains may interact with paxillin, Nck-interacting kinase (NIK), or WASP [7-9,13].

Neurons contain numerous scaffold proteins that are involved in the regulation of synaptic function [14]. Cask-interactive proteins (Caskins) also belong to this group of scaffold proteins [15]. Caskin1 and its isoform Caskin2 are multidomain proteins possessing six ankyrin repeats, a single SH3 domain, and two sterile α motifs (SAM domains) in the N-terminal part [15]. In contrast, there are no recognizable domains in the C-terminal part, which is dominated by a long proline-rich region [15]. Recently, we provided evidence that the entire proline-rich region of Caskin1 is intrinsically disordered. We also showed that the proline-rich region is biochemically functional, as it interacts with the adaptor protein Abl-interactor-2 (Abi2) [16]. In addition to CASK and Abi2, Caskin1 can also interact with Nck/Dock, neurexin2, synaptotagmin, and septin4 [12,16,17]. A Caskin homolog has been recently discovered in *Drosophila* and shown to be involved in LAR receptor tyrosine phosphatase-mediated motor axon guidance [17]. Recent data suggest that the tandem SAM domains of Caskin1 form a novel SAM polymer containing eight SAM domains per helical turn with an unusually long helical pitch. This special feature of Caskin1 may also contribute to the organization of synaptic function [18].

Here we show that receptor tyrosine kinase EphB1 can form a complex with Caskin1 through the adaptor protein Nck. Upon activation of the receptor kinase, the SH2 domain of Nck binds to one of its tyrosine residues, while the Nck SH3 domains interact with the proline-rich domain of Caskin1. Complex formation of the receptor kinase, adaptor, and scaffold proteins results in the phosphorylation of tyrosine residues 296 and 336 localized in the SH3 domain of Caskin1. Using CD spectroscopy we found that upon tyrosine phosphorylation the structure of the Caskin1 SH3 domain changes significantly.

## Results

### Nck interacts with Caskin1 in an SH3 domain-dependent manner

**Figure 1:**
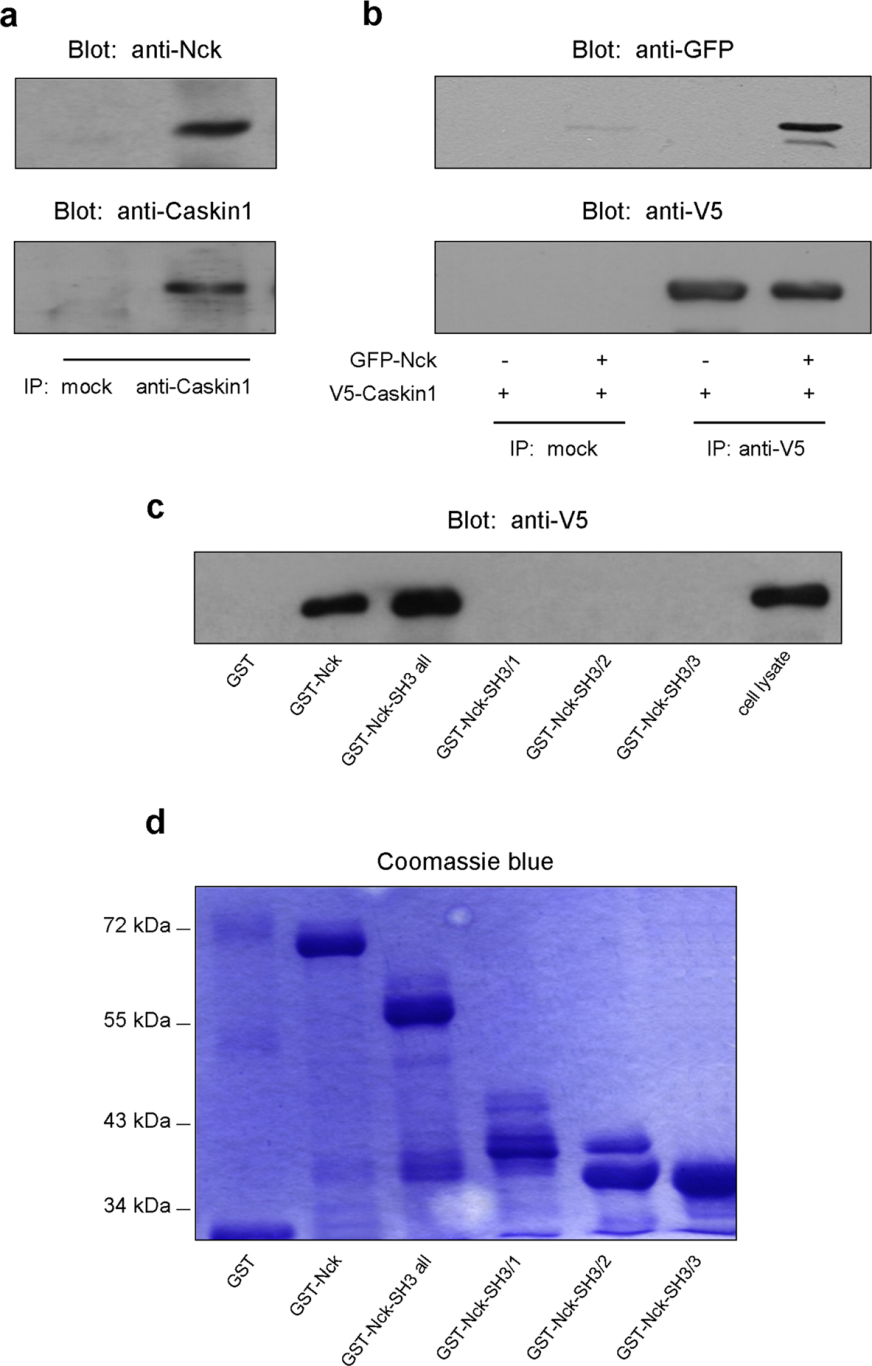
**Nck interacts with Caskin1 through its SH3 domains.** (**a**) Endogenous Caskin1 was immunoprecipitated (IP) from rat brain lysate with a monoclonal anti-Caskin1 antibody. After SDS-PAGE and transfer to nitrocellulose, samples were analyzed by anti-Nck and anti-Caskin1 antibodies. (**b**) V5 epitope-tagged Caskin1 and/or GFP-tagged Nck were transiently expressed in COS7 cells, and then cell lysates were immunoprecipitated with an anti-V5 antibody. Immunoprecipitates were immunoblotted with anti-GFP and anti-V5 antibodies. (**c**) Full length Nck, the three SH3 domains together (Nck-SH3-all), and individual SH3 domains (SH3/1, SH3/2 and SH3/3) of Nck were generated as GST fusion proteins. These fusion proteins were immobilized on glutathione-agarose beads and used for protein precipitations from lysates of COS7 cells expressing V5-Caskin1. Precipitated proteins were separated on SDS-PAGE and transferred to nitrocellulose, and then samples were immunoblotted with anti-V5 antibody. (**d**) Coomassie stained gels with GST fusion proteins used in the previous experiment. These results are representative of three experiments.

We and others have shown earlier that the scaffold protein Caskin1 interacts with Nck/Dock [8,12,16,17]. To confirm and characterize the interaction between the two proteins, first, endogenous Caskin1 was immunoprecipitated (IP) from rat brain lysate with a monoclonal anti-Caskin1 antibody. After SDS-PAGE and transfer to nitrocellulose, samples were analyzed by anti-Nck and anti-Caskin1 antibodies. As Figure 1a shows Caskin1 interacts stably with Nck *in vivo*. To study the interaction in another system, V5 epitope-tagged Caskin1 and/or GFP-tagged Nck were transiently expressed in COS7 cells, and then cell lysates were immunoprecipitated with an anti-V5 antibody. When both proteins were coexpressed in cells, Caskin1 was capable of associating with Nck (Figure 1b). Nck possesses an SH2 and three SH3 domains [6]. Although it has been shown earlier that the interaction between Caskin1 and Nck is mediated through SH3 domains [12], it has not been determined which SH3 domain(s) is responsible for the association. Therefore, to determine the required SH3 domains for the Caskin1/Nck interaction, full length Nck, the three SH3 domains together (Nck-SH3-all), and individual SH3 domains (SH3/1, SH3/2 and SH3/3) were generated as GST fusion proteins. These fusion proteins were immobilized on glutathione-agarose beads and used for protein precipitations from lysates of COS7 cells expressing V5-Caskin1. As seen in Figure 1c, none of the individual SH3 domains of Nck was able to pull-down the scaffold protein. However, when all three SH3 domains were combined, either in the full length Nck or the Nck-SH3-all construct, Caskin1 was readily precipitated. These findings suggest that similar to many of the Nck-interacting proteins (described in the Discussion) Caskin1 requires all SH3 domains of Nck for high affinity interaction. Figure 1d demonstrates that the majority of the Nck GST fusion proteins gave single bands after SDS-PAGE and Commassie-blue staining.

### Caskin1 is recruited to the EphB1 tyrosine kinase via Nck

**Figure 2:**
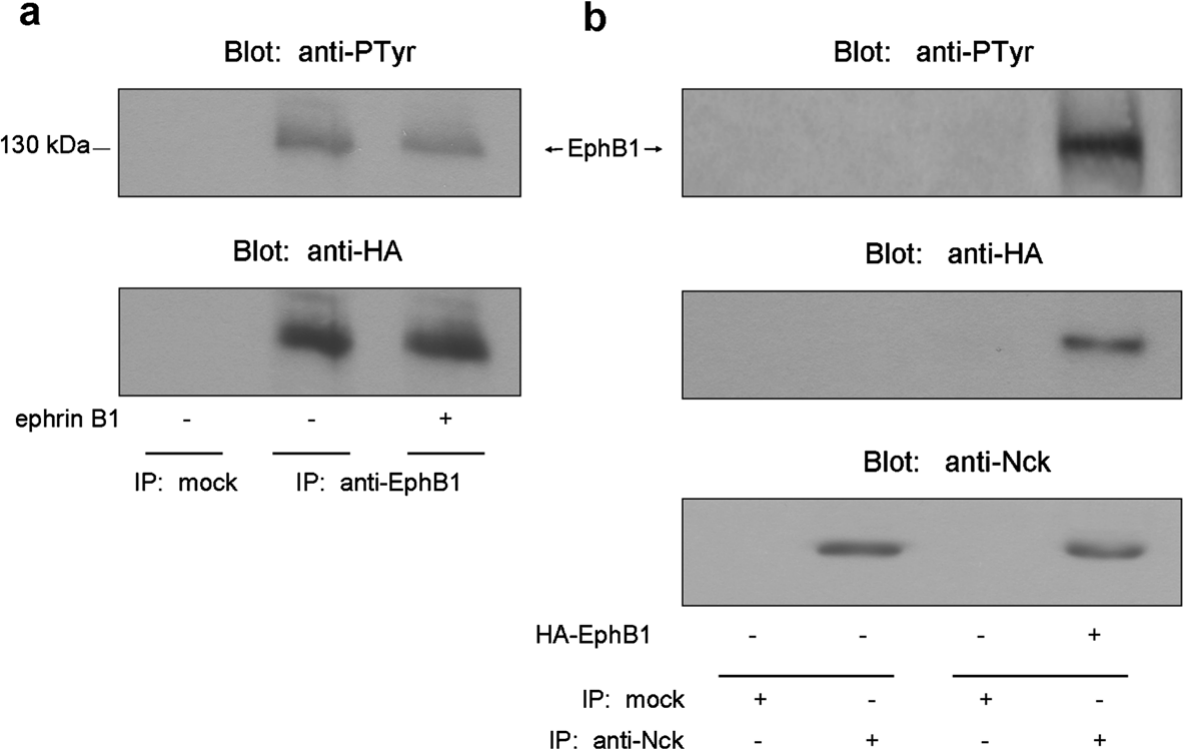
**Expression of EphB1 in COS7 cells results in autoactivation and interaction with Nck.** (**a**) COS7 cells were transiently transfected with HA-tagged EphB1 and after overnight serum-starvation cells were stimulated with ephrinB1 for 20 minutes or left untreated. Proteins were immunoprecipitated with anti-EphB1 antibody and probed with anti-phosphotyrosine and anti-HA antibodies. (**b**) HA-tagged EphB1 was transiently expressed in COS7 cells. Cell lysates were then subjected to immunoprecipitation with an anti-Nck antibody. Bound proteins were separated by SDS-PAGE, transferred to nitrocellulose, and probed with anti-phosphotyrosine, anti-HA, and anti-Nck antibodies. These results are representative of three experiments.

**Figure 3:**
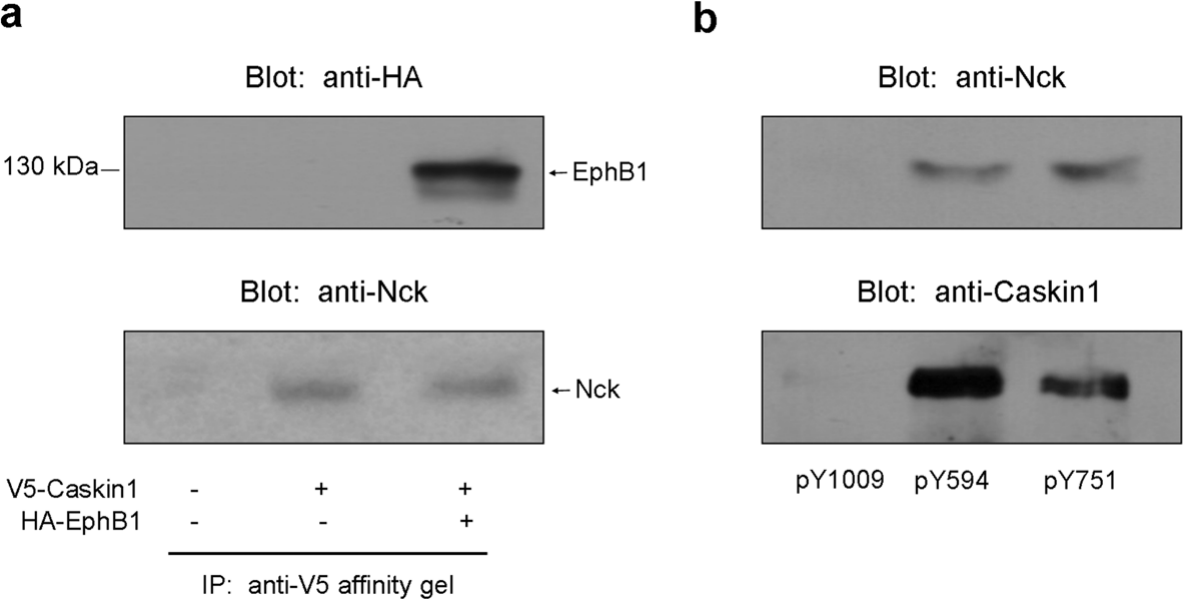
**Caskin1 is recruited to EphB1 tyrosine kinase via Nck.** (**a**) COS7 cells were transiently transfected with HA-EphB1 or/and V5-Caskin1, cell lysates were then subjected to immunoprecipitation with anti-V5 antibody. Bound proteins were separated by SDS-PAGE, transferred to nitrocellulose, and probed with anti-HA and anti-Nck antibodies. (**b**) Biotinylated tyrosine phosphopeptides were synthesized (Y594-P, Y751-P, and Y1009-P as negative control) and immobilized on streptavidin-beads as described in the Methods. These phosphopeptides were incubated with rat brain lysates. Precipitated proteins were resolved by SDS-PAGE, transferred to nitrocellulose and analyzed by anti-Caskin1 and anti-Nck antibodies.

It has been well documented that a number of tyrosine kinases or their substrates can recruit Nck through its SH2 domain [6]. One of the tyrosine kinase receptors present in the nervous system is EphB1 which was reported to bind to Nck through its phosphorylated Tyr-594 residue [7]. Therefore, we hypothesized that Nck could mediate an interaction between EphB1 and Caskin1. To address this question, first, HA-tagged EphB1 was transiently expressed in COS7 cells stimulated with ephrin B1 the ligand of the receptor tyrosine kinase. As Figure 2a demonstrates expression of EphB1 alone in these cells induces activation of the kinase which is reflected in its tyrosine phosphorylation. Interestingly, addition of the ligand did not further increase the receptor tyrosine phosphorylation. Other groups also found that expression of EphB1 or EphA3 in cells leads to autoactivation of these receptor kinases [11,19]. Next, we tested if Nck could bind to EphB1 in COS7 cells expressing the HA-tagged kinase. It was found that an antibody against the endogenous Nck could coimmunoprecipitate EphB1 tyrosine kinases (Figure 2b). To prove the existence of the EphB1/Nck/Caskin1 complex, HA-EphB1 and V5-Caskin1 were transiently coexpressed in COS7 cells, and then cell lysates were immunoprecipitated with an anti-V5 antibody covalently bound to Sepharose beads. As seen in Figure 3a, Caskin1 immunoprecipitates both Nck and EphB1.To prove in a more physiological context that a specific phosphotyrosine residue in EphB1 may recruit the complex of Nck/Caskin1, we used phosphopeptides immobilized on beads to precipitate proteins, as described earlier [20,21]. The SH2 domain of Nck1 is known to bind to the EphB1 autophosphorylation site Y594 and to the PDGF receptor autophosphorylation site Y751 [7,22]. Therefore, two phosphopeptides containing the aforementioned phosphorylation sites were synthesized (Y594-P and Y751-P). A control phosphopeptide was also synthesized based on the autophosphorylation site Y1009 of the PDGF receptor that cannot bind to Nck1 [7]. Figure 3b demonstrates that both Nck-specific phosphopeptides, Y594-P and Y751-P, immobilized on Streptavidin-agarose resin were capable of precipitating the complex of Nck and Caskin1 from rat brain lysates, while the control phosphopeptide was not able to pull-down the proteins. These experiments strongly suggest that the activated and autophosphorylated EphB1 receptor may recruit the complex of Nck/Caskin1 in neuronal cells.

### EphB1 phosphorylates Caskin1 on tyrosine 296 and 336

**Figure 4:**
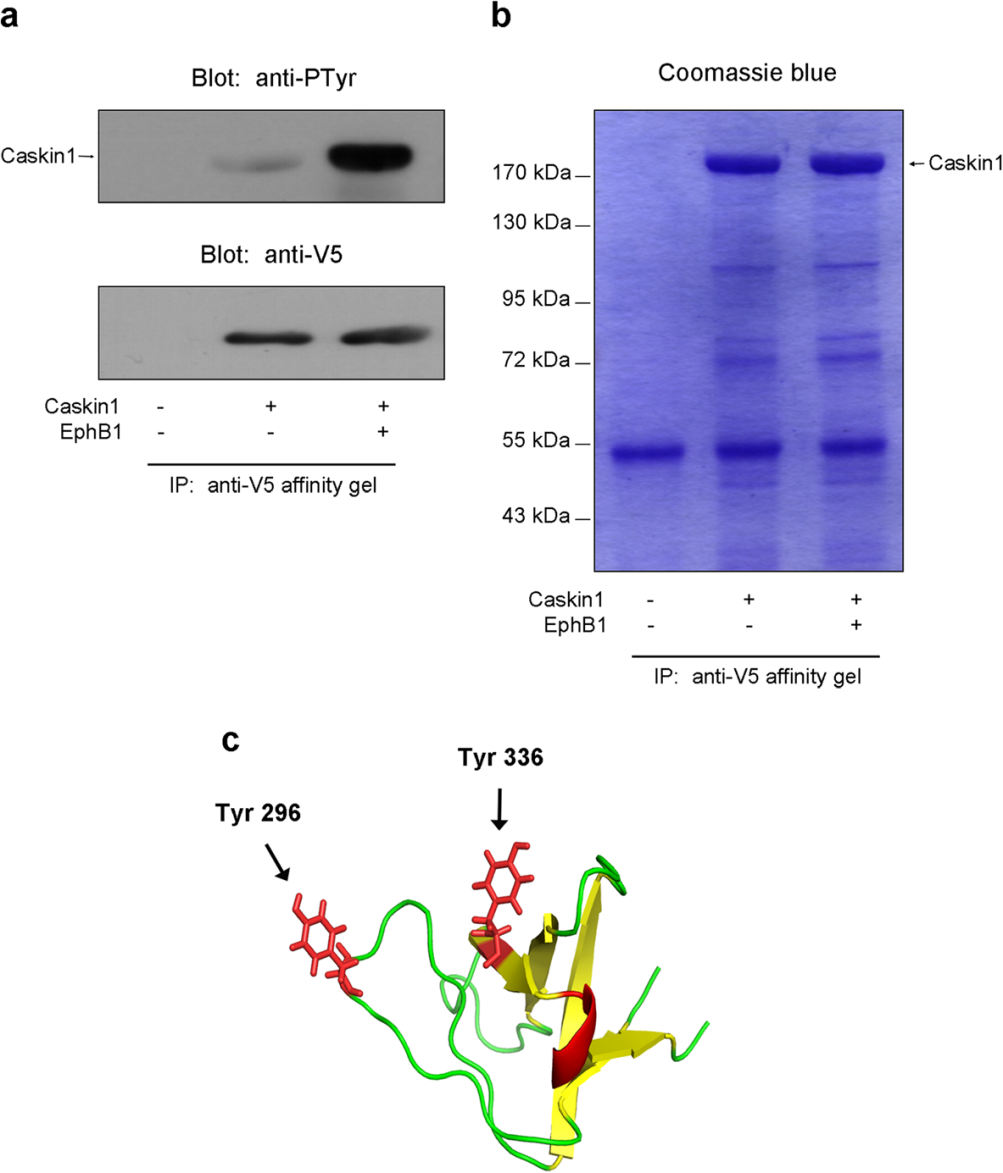
**EphB1 phosphorylates Caskin1 on tyrosine 296 and 336(1).** (**a**) V5-Caskin1 and HA-EphB1 were transiently coexpressed in COS7 cells. Cell lysates were then subjected to immunoprecipitation with anti-V5 antibody. Bound proteins were separated by SDS-PAGE, transferred to nitrocellulose, and probed with anti-phosphotyrosine and anti-V5 antibodies. (**b**) V5-Caskin1 and HA-EphB1 were transiently coexpressed in COS7 cells as in Figure 4a, however, after immunoprecipitation with anti-V5 antibody protein samples were stained by Coomassie blue. The position of the bands sent to mass spectrometry analysis (Caskin1) is indicated. (**c**) The 3D structure of the rat Caskin1 SH3 domain was modeled using the public I-TASSER ( http://zhanglab.ccmb.med.umich.edu/I-TASSER) structure prediction server. The positions of the two tyrosines phosphorylated by EphB1 are indicated.

It has been well established that receptor tyrosine kinases bind signaling molecules and often phosphorylate them [23]. Therefore, we hypothesized that Caskin1 recruited to the activated EphB1 receptor tyrosine kinases could be subject to tyrosine phosphorylation. To challenge our hypothesis, Caskin1 and EphB1 were transiently coexpressed in COS7 cells and then tyrosine phosphorylation of Caskin1 was tested. As shown in Figure 4a, coexpression of Caskin1 with EphB1 resulted in a significant tyrosine phosphorylation of the scaffold protein. We used mass spectrometry to determine the site(s) of Caskin1 phosphorylation. To this end, Caskin1 was transiently expressed alone or together with EphB1 in COS7 cells and immunoprecipitated with anti-V5 antibody. After SDS-PAGE and staining with Coomassie blue, protein bands corresponding to unphosphorylated and EphB1-phosphorylated Caskin1 (Figure 4b) were cut out from the gel and sent to mass spectrometry analysis. Two tyrosine residues were identified to be phosphorylated in Caskin1: Y296 and Y336 (Additional file 1). Interestingly, both tyrosine residues are located within the SH3 domain. To determine the position of the phosphorylated tyrosine residues, the 3D structure of the rat Caskin1 SH3 domain was modeled by using the public I-TASSER structure prediction server as described in Methods. As shown in Figure 4c, Tyr296 is localized in the flexible RT loop linking the first two beta-strands, while Tyr336 is present in the more compact fourth beta-strand.

**Figure 5:**
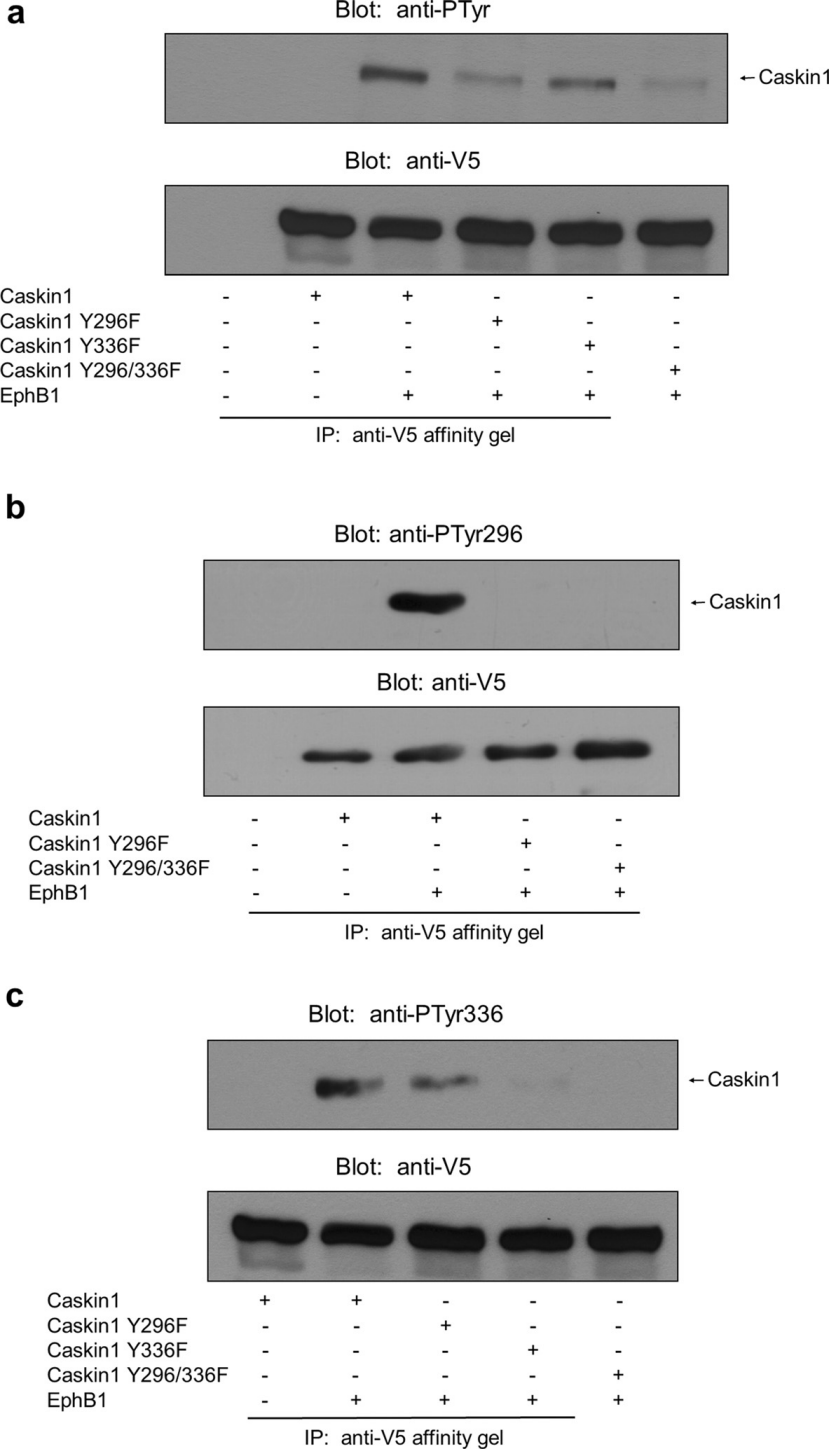
**EphB1 phosphorylates Caskin1 on tyrosine 296 and 336(2).** (**a**) Ha-EphB1 and different constructs of V5-Caskin1 were coexpressed in COS7 cells. Cell lysates were then subjected to immunoprecipitation with anti-V5 antibody. Bound proteins were separated by SDS-PAGE, transferred to nitrocellulose, and probed with anti-phosphotyrosine and anti-V5 antibodies. (**b**) HA-EphB1 and different constructs of V5-Caskin1 were coexpressed in COS7 cells. Cell lysates were then subjected to immunoprecipitation with anti-V5 antibody. Bound proteins were separated by SDS-PAGE, transferred to nitrocellulose, and probed with the phosphospecific anti-PTyr296 and anti-V5 antibodies. (**c**) HA-EphB1 and different constructs of V5-Caskin1 were coexpressed in COS7 cells. Proteins were then immunoprecipitated with anti-V5 antibody, then samples were analyzed by the phosphospecific anti-PTyr336 and anti-V5 antibodies.

To show definitively that Y296 and Y336 are tyrosine phosphorylated, phosphospecific antibodies were generated (anti-PTyr296 and anti-PTyr336). In addition, point mutations were introduced into the SH3 domain of Caskin1, changing tyrosine 296 (Y296F) and tyrosine 336 (Y336F), respectively, or both tyrosine 296 and 336 (Y296/336F) to phenylalanines. Caskin1 together with EphB1 were then transiently coexpressed in COS7 cells and tyrosine phosphorylation of Caskin1 was probed with the anti-phosphotyrosine antibody 4G10 or with the phosphospecific antibodies. Figure 5a demonstrates that, as shown in Figure 4a, wild type Caskin1 is tyrosine phoshorylated in the presence of EphB1. However, both the Caskin1 Y296F and Y336F constructs showed markedly reduced phosphorylation levels. The lowest level of phosphorylation was detected when both tyrosine residues (Y296/336F) were mutated (Figure 5a). Next, anti-PTyr296 and anti-PTyr336 antibodies were tested under similar conditions. Tyrosine phosphorylation of Caskin1 was unambiguously detected with the anti-PTyr296 antibody which signal was completely abolished when Y296F or Y296/336F constructs were used (Figure 5b). The phosphospecific anti-PTyr336 antibody also clearly detected the phosphorylation of wild type Caskin1. This phosphorylation level was decreased when the either the Y336F or the Y296/336F constructs were applied. Taken together, these results suggest that Caskin1 when recruited to EphB1 tyrosine kinase is phosphorylated on tyrosine residues 296 and 336.

### Tyrosine phosphorylation leads to structural changes in the Caskin1 SH3 domain

**Figure 6:**
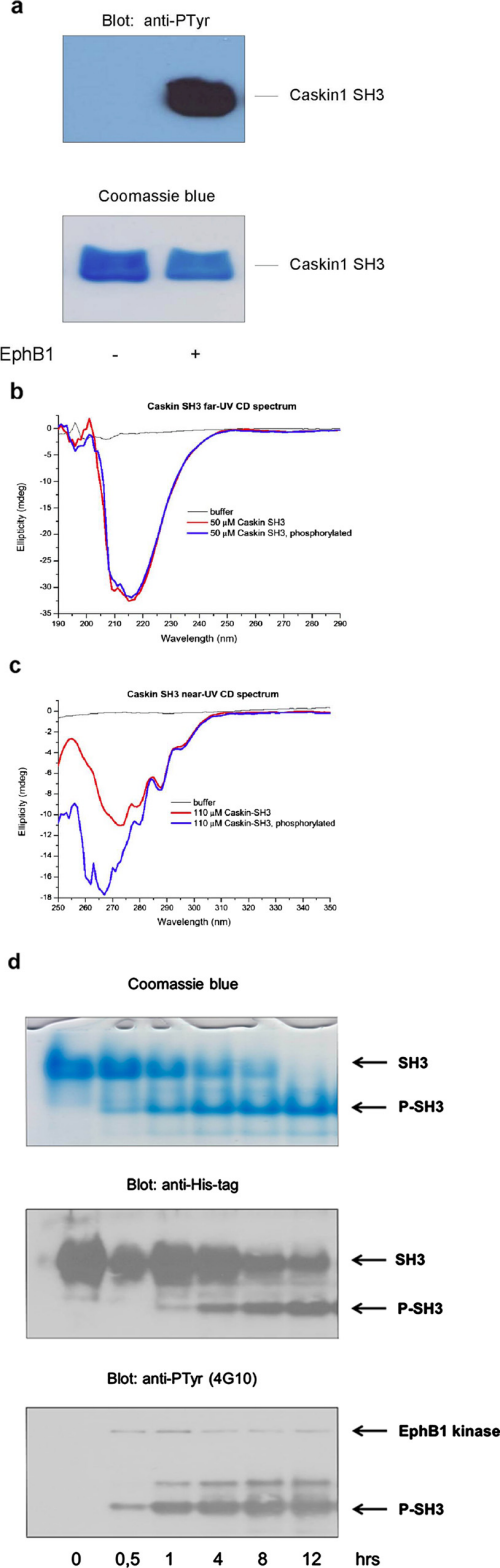
**Tyrosine phosphorylation leads to structural changes in the Caskin1 SH3 domain.** (**a**) The SH3 domain of Caskin1 was expressed, purified, and phosphorylated in vitro by EphB1 as described in the Methods. The upper panel demonstrates the phosphorylation of the SH3 domain probed with anti-phosphotyrosine antibody. The lower panel shows the protein samples stained with Coomassie blue. (**b**) Far-UV CD spectra were measured of the unphosphorylated and the phosphorylated SH3 domains. The CD spectrum of the buffer itself is also indicated. (**c**) Near-UV CD spectra were measured of the unphosphorylated and the phosphorylated SH3 domains. The CD spectrum of the buffer itself is also indicated. These results are representative of three experiments. (**d**) The SH3 domain of Caskin1 was expressed, purified, and phosphorylated in vitro by EphB1 as described in the Methods. The upper panel demonstrates the time course of phosphorylation of the SH3 domain stained with Coomassie-blue. After blotting the proteins to nitrocellulose membrane, immunoblots were performed using anti-His-tag and anti-phosphotyrosine antibodies (middle and lower panels).

Recently, it has emerged that tyrosine phosphorylation of SH3 domains may negatively regulate protein-protein associations. It was proposed that the phosphotyrosine residue(s) and its charge may interfere with binding of polyproline helices of SH3 domain interacting partners [24]. However, the potential structural changes caused by the phosphorylation have not been determined experimentally so far. Therefore, we expressed and purified the Caskin1 SH3 domain and phosphorylated it *in vitro* by a recombinant active EphB1. Figure 6a demonstrates that under the applied conditions the SH3 domain is intensively phosphorylated on tyrosine residues. Next, far-UV CD spectrum was measured which can reveal important characteristics of the secondary structure of a protein. As shown in Figure 6b, no major difference was detected between the spectra of unphosphorylated and phosphorylated SH3 domains suggesting that phosphorylation of tyrosine residues does not cause significant changes in the secondary structure. Interestingly, when near-UV CD spectra were taken noticeable changes were detected upon tyrosine phosphorylation of the SH3 domain. It seems that the chemical environment of these residues is clearly sensitive to phosphorylation suggesting that tertiary structure of the SH3 domain is likely altered around the phosphorylated tyrosines. However, it is also possible that there are associated quaternary structural changes, e.g. dimerization, that could alter the spectrum. To exclude this possibility, time-course of Caskin1 phosphorylation was followed at 15°C by using native gel electrophoresis. Figure 6d demonstrates that tyrosine phosphorylated Caskin1 SH3 domain migrates faster than the unphosphorylated protein, possibly due to the incorporated negative charges. Nevertheless, the native gel electrophoresis does not show any sign of protein dimerization or aggregation induced by EphB1-dependent phosphorylation.

## Discussion

Intracellular signaling pathways mediating the biological effects of ephrins and their receptors have been intensively characterized in the recent years. Although a number of proteins binding to EphB receptor kinases have been identified, our knowledge of their function particularly in axonal guidance is still incomplete. Here, we describe a novel pathway in which the neuron-specific scaffold protein Caskin1 forms a complex with the EphB1 receptor via the Nck adaptor protein. Previously, it has been shown that Caskin1 could bind to several partners, including Cask, Nck/Dock, LAR receptor proteins tyrosine phosphatase, synaptotagmin, Abi2, and neurexin [15-17]. However, it is still unclear which receptor or transmembrane proteins could regulate Caskin1 function.

**Figure 7:**
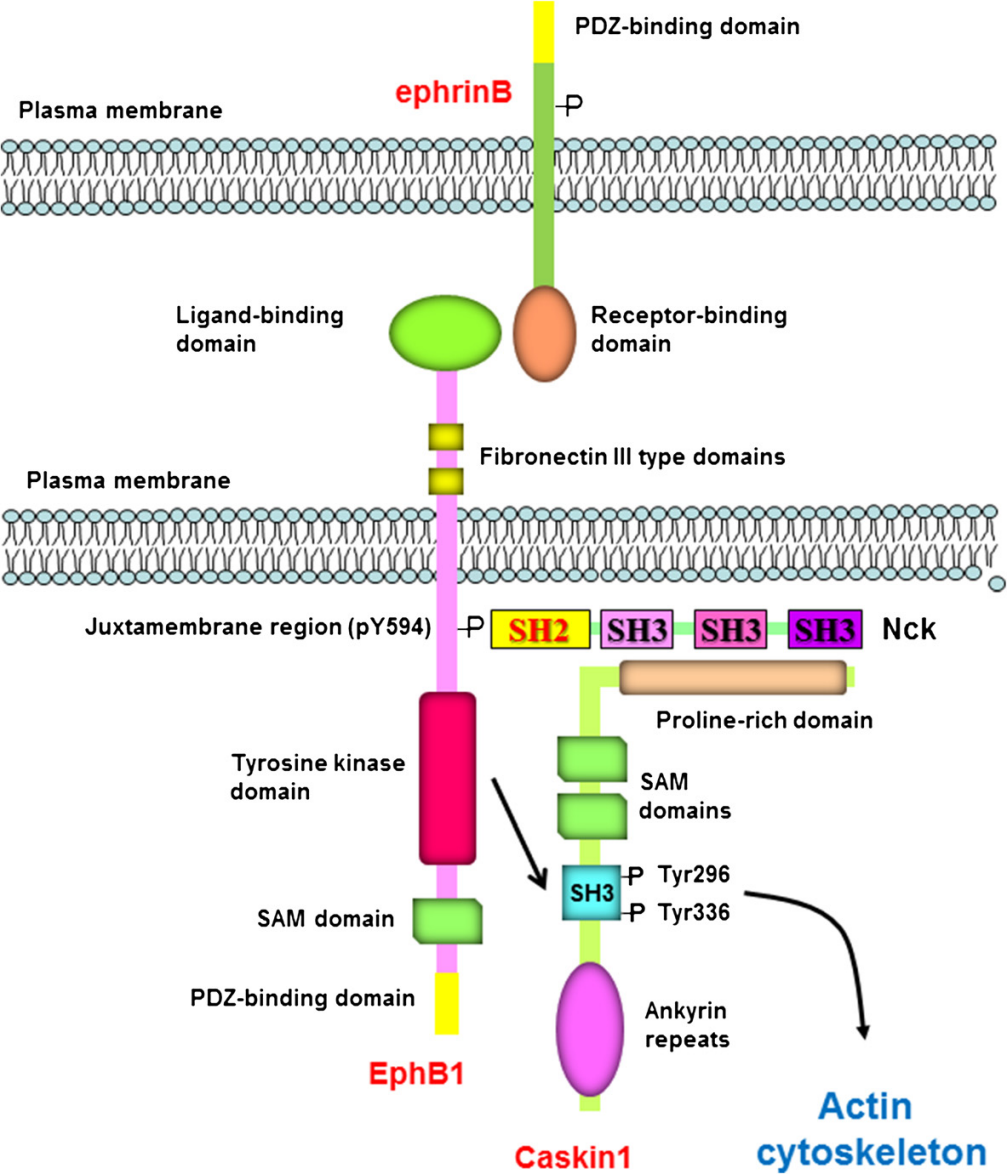
**Proposed model of the EphB1**-**Caskin1 interaction.** Ligand-activated EphB1 recruits the adaptor protein Nck through the phosphorylated Y594 residue. In turn, Nck forms a complex with Caskin1 via the SH3 domains of Nck. Recruitment of Caskin1 to the vicinity of the receptor leads to the phosphorylation of the Caskin1 SH3 domain on tyrosine residues 296 and 336. Tyrosine phosphorylated Caskin1 then likely promotes reorganization of the actin cytoskeleton leading to spine formation. PDZ, (**p**ostsynaptic density 95, PSD-85; discs large, **D**lg; zonula occludens-1, **Z**O-1), SH2, Src-homology domain 2, SH3, Src-homology domain 3, SAM, sterile alpha motif.

Interaction of EphB1 with Nck/Dock has been studied intensively [7,12,16,17]. Tyrosine 594 within the juxtamembrane region of EphB1 was shown to be required for binding the SH2 domain of Nck [7], while its SH3 domains may interact with paxillin, Nck-interacting kinase (NIK), and WASP, participating in the regulation of actin cytoskeleton [7-9,13]. Association of EphB1 kinase with Caskin1 through Nck represents the fourth possibility of how EphB1 may signal toward actin cytoskeleton (Figure 7). Although the interaction of Nck with EphB1 is well established, much less is known about the interaction of Nck SH3 domains with downstream partners. Therefore, we wished to determine which of the SH3 domains of Nck was required for the interaction. It seems that none of the individual SH3 domains of Nck is able to pull-down Caskin1. Instead, all three SH3 domains are necessary for the binding. This finding is not surprising since many of the binding partners of Nck require the presence of two or three SH3 domains for high affinity binding [6,25]. This comes from the fact that the affinity of the SH3 domains for their ligands ranges typically from low micromolar to mid-nanomolar [26-28]. Therefore, SH3 domain containing proteins apply diverse strategies to increase the strength of the interaction: they contain multiple SH3 domains, such as Grb2, Nck or the Tks family of scaffold proteins, or use additional surfaces for ligand binding on the SH3 domain outside the PPII binding groove, such as the highly variable RT and n-Src loops [26,27].

Complex formation of EphB1 with Caskin1 resulted in the phosphorylation of tyrosine residues 296 and 336 (Figure 7). Identification of these sites was somewhat surprising as both tyrosines are localized within the SH3 domain. By modeling the 3D structure of the rat Caskin1 SH3 domain it was found that Tyr296 is localized in the flexible RT loop linking the first two beta-strands, while Tyr336 is present in the more compact fourth beta-strand. Recent evidence suggests that several SH3 domains can be subject of phosphorylation on conserved tyrosine residues [24]. For example, the C-terminal SH3 domain of Grb2 can be phosphorylated by EGF receptor or Bcr/Abl on Y209 [29]. This phosphorylation resulted in reduced binding of proline-rich peptides based on the structure of the Sos exchange protein [29]. Phosphorylation of Abi1 adaptor protein by Abl also decreases binding of Abi1 to Abl kinase [30]. Unfortunately, in the literature there are no identified binding partners for the SH3 domain of Caskin1. Therefore, we could not test if phosphorylation of Caskin1 SH3 domain interferes with its ligand binding. However, we investigated if the phosphorylation could have any effect on the structure of the protein. Near-UV CD spectra were taken which showed noticeable changes upon tyrosine phosphorylation of the SH3 domain. It seems that the chemical environment of these residues is clearly sensitive to phosphorylation, suggesting that the tertiary structure of the SH3 domain is likely altered around the phosphorylated tyrosines. Nevertheless, it is also possible that pY296 and pY336 may represent binding sites for SH2 domain-containing signaling molecules thereby positively regulating the interactions of Caskin1.

Tandem mass spectrometry analysis of mouse postsynaptic density and synaptosomal preparations revealed several serine/threonine phosphorylation sites on the proline-rich region of Caskin1 [31,32]. Recently, a study using kinase-selective enrichment for quantitative phosphoproteomics identified Y336 in Caskin1 as an *in vivo* phosphorylation site [33]. This independent work confirms that, at least, Y336 is a physiological phosphorylation site that might have a regulatory role in the function of Caskin1. Previous data suggested that cell surface EphB receptors are likely molecules that transduce extracellular into the dendrite to trigger spine morphogenesis [34,35]. The role of EphB receptors in spine development is in line with previous reports indicating that EphB receptors can be found in the post-synaptic structures of dendrites [36,37]. In addition, another group has shown that EphB receptor kinases contribute to dynamic reorganization of the action cytoskeleton leading to spine morphological features, such as filopodia shrinkage and formation of stable, mature, mushroom-like spines [38]. Altogether, these and our data suggest that EphB receptors recruiting the complex of Nck and Caskin1 to the plasma membrane may also contribute to the cytoskeletal organization of dendritic spines (Figure 7). Nevertheless, further experiments will be required to better understand the function and regulation of Caskin1 in the nervous system.

## Conclusions

In summary, we show here that the scaffold protein Caskin1 can be recruited to the activated and autophosphorylated EphB1 tyrosine kinase via the Nck protein as a linker. Complex formation results in tyrosine phosphorylation of Caskin1 SH3 domain on tyrosine residues 296 and 336. Although we were not able to identify any physiological partners of the Caskin1 SH3 domain so far, we could demonstrate by near-UV CD spectrometry that phosphorylation on conserved tyrosine residues results in marked changes in the structure of the SH3 domain.

## Methods

### Antibodies, constructs and reagents

The monoclonal antibody raised against GFP was a kind gift of Cancer Research UK Hybridoma Development Unit, London, UK. Polyclonal anti-Caskin1 antibody was generated against the SAM domains of Caskin1. Monoclonal anti-Caskin1 antibody was custom-made by AbDSerotec. Monoclonal antibody against Nck (610009) was from BD Transduction Laboratories. Antibodies against Nck (polyclonal, 06–288) and the phosphotyrosine residues (clone 4G10, 05–321) were obtained from Millipore (Billerica, MA). Antibodies against the V5 epitope (R96025 and A7345) were purchased from Invitrogen (Carlsbad, CA) and Sigma-Aldrich (St. Louis, MO), respectively. Anti-HA monoclonal antibody (6E2, 2367) was from Cell Signaling Technologies (Beverly, MA). Anti-EphB1 polyclonal antibody (Q-20, sc-926) was purchased from Santa Cruz Biotechnology, Inc. Anti-pTyr296 (DYCNN{pTyr}DLTSLN) and anti-pTyr336 (GNDRVG{pTyr}FPSSLGC) phosphospecific polyclonal antibodies were custom-synthesized by Genscript (Piscataway, NJ).

The full length HA-tagged EphB1 was kindly provided by Uyen Huynh-Do (University of Bern Medical School, Bern, Switzerland). The full length rat Caskin1 cDNA was kindly provided by Thomas Südhof (University of Texas Southwestern Medical Center, Dallas, TX, USA). Caskin1 cDNA was amplified by a high-fidelity DNA polymerase and subcloned into the pcDNA 3.1 / V5-His TOPO vector (Invitrogen, San Diego, CA, USA). GFP-Nck and different domain constructs of Nck expressed as GST fusion proteins were described earlier [25,39]. For pull-down experiments, GST fusion proteins were purified by binding to glutathione-agarose (Sigma-Aldrich). Protein purification was monitored on Coomassie blue-stained SDS-PAGE gels: the majority of the GST proteins gave single bands. The DNA fragment encoding the rat Caskin1 SH3 domain was amplified by Pfu Turbo HotStart DNA polymerase (Agilent) from full-length rat Caskin1 cDNA by PCR and subcloned into the NdeI/XhoI sites of expression vector pET22b (New England Biolabs). EphB1-pY594 (PQMKI{pTyr}IDPFTK), PDGFR-pY751 (DESVD{pTyr}VPMLDK), and PDGFR-Y1009 (TSSVL{pTyr}TAVQPK) biotinylated phosphopeptides and the corresponding phosphospecific antibodies were produced by Invitrogen. V5-Caskin1Y296F, V5-Caskin1Y336F, and V5-Caskin1Y296/336F mutants were generated using the QuickChange Site-directed Mutagenesis Kit (Stratagene, La Jolla, CA). In all cases the constructs were verified by DNA sequencing. Stock solutions of ephrin B1-Fc (473-EB-200, R&D Systems) was prepared according to the manufacturer’s instructions. Streptavidin-agarose (S1638) and active recombinant EphB1 tyrosine kinase (E7032) were purchased from Sigma-Aldrich.

### Cell lines, transfection, and stimulation

COS7 cells were purchased from American Type Culture Collection and maintained in Dulbecco’s modified Eagle’s medium (DMEM) supplemented with 10% fetal calf serum (Invitrogen), penicillin (100 units/ml), and streptomycin (50 μg/ml). Cells were transiently transfected with Lipofectamine (Invitrogen) according to the manufacturer’s instructions. For stimulation, cells were serum-starved overnight and stimulated with ephrin B1-Fc at 500 ng/ml for 20 min.

#### SDS-PAGE and immunoblotting

SDS-PAGE and immunoblotting was performed as described previously [40]. Briefly, COS7 cells were washed with ice-cold PBS and lysed in 1 ml of ice-cold 50 mM Hepes buffer, pH 7.4, containing 100 mM NaCl, 1% Triton X-100, 20 mM NaF, 1 mM EGTA, 1 mM Na_3_VO_4_, 1 mM p-nitrophenyl-phosphate, 10 mM benzamidine, 1 mM phenylmethylsulphonyl fluoride, 25 μg/ml each of leupeptin, soybean trypsin inhibitor and aprotinin. Lysates were clarified by centrifugation at 15.000 × g for 10 min at 4°C and then incubated for 1 h at 4°C with 30 μl of anti-V5 agarose affinity gel (A7345, Sigma-Aldrich), or monoclonal anti-HA agarose (A2095, Sigma-Aldrich), or 5 μl of monoclonal anti-Caskin1 antibody and 25 μl of Protein G Sepharose beads (GE Healthcare), or 5 μl of polyclonal anti-Nck and 25 μl of Protein A Sepharose beads (Sigma-Aldrich), or with 5 μg of GST fusion proteins immobilized on glutathione-agarose beads (GST pull-down experiments). Protein precipitates were washed three times with ice-cold PBS, pH 7.4, containing 0.4% Triton X-100 and eluted with SDS sample buffer. Proteins were then separated by SDS-PAGE, transferred to nitrocellulose membrane and immunoblotted with the indicated antibodies. Blots were developed by the enhanced chemiluminescence (ECL, Amersham Pharmacia Biotech) system.

#### In vitro phosphorylation

The kinase reaction mixture (310 μl) included purified Caskin1 SH3 domain (200 μl, 3.8 mg/ml), a 3-fold kinase buffer (100 μl - 200 mM Tris buffer, pH 7.5, 20 mM MgCl_2_) and 10 μl recombinant, GST-tagged EphB1 receptor (E7032, Sigma-Aldrich). The phosphorylation was initiated with 7.5 μl 10 mM ice-cold ATP. The control reaction mixture did not include ATP. The incubation was carried out at 30°C for 1 h. For native gel electrophoresis, the phosphorylation of Caskin1 was performed as above, with the exception that the incubation was carried out at 15°C for the indicated times.

#### Purification of Caskin1 SH3 domain

For structural characterization, the 6x His-tagged SH3 protein was expressed in the E. coli strain BL21 star. Cells were grown in 500 ml NZYM medium at 37°C, 250 rpm up to an OD600 of 0.5-0.6. Expression of the protein was induced by 0.5 mM isopropyl thio-β-D-galactoside (Duchefa) at 20°C for 5 h. Cells were harvested by centrifugation for 20 minutes at 4°C and 4000 rpm then lysed in 10 ml binding buffer (20 mM sodium phosphate pH 7.4, 500 mM NaCl and 20 mM imidazole). After sonication for 10 seconds 10 times, the cell lysate was treated with 1% Triton X-100 for 30 minutes on ice. The cell debris was removed by centrifugation for 40 minutes at 4°C and 50.000 rpm and the supernatant was subsequently cleared by using a 0.20 μM filter. The protein was purified in AKTA Explorer Protein Purification System (GE Healthcare) supplied with a 1 ml HisTrap HP column (GE Healthcare). The loading of the cleared lysate onto the pre-equilibrated column was followed by a 10 column volume washing step using the binding buffer. The target protein was eluted with the same buffer containing 1 M imidazole using a step elution protocol. Fractions containing the 6xHis tagged Caskin1 SH3 domain were applied to an AKTA HiPrep 26/10 Desalting column (GE Healthcare) to remove imidazole and to change the buffer to the appropriate one. The purity of the protein was monitored by SDS-PAGE.

#### Phosphorylation site mapping

Gel bands were cleaned-up with in-gel reduction (10 mM DTT) and alkylation (50 mM IAA) prior to in-gel digestion with trypsin. In-gel reduction/alkylation: 10 mM DTT in 100 mM ammonium bicarbonate was added to gel bands and incubated at 56°C for 60 mins, the buffer was then discarded. Next, 50 mM IAA in 100 mM ammonium bicarbonate was added to gel bands and incubated at room temperature in the dark for 30 mins, the buffer was then discarded. Gel bands were then washed in 50:50 ammonium bicarbonate (20 mM): acetonitrile, then 100% acetonitrile, (all 15 mins – buffer s then discarded). Gel bands were dried in speed vac. In-gel trypsin digestion: stock trypsin (Roche modified sequencing grade) was prepared at 1 mg/ml concentration in 1 mM HCL and stored at −20°C until required. This stock solution was adjusted to 12.5 ug/ml in 20 mM ammonium bicarbonate and added to dry gel bands – just enough to rehydrate gel bands and cover. Digests were then incubated overnight at 30°C on shaking platform. The next day an equal volume of acetonitrile was added and incubated for 20 mins.

##### Phosphopeptide enrichment

Strong cation exchange (SCX) fractionation of tryptic peptides was performed on a 3.0-mm × 20-cm column (Poly LC, Columbia, MD) containing 5-μm polysulfoethyl aspartamide beads with a 200-Å pore size. The first 15 SCX fractions were freeze dried and subjected to titanium dioxide (TiO_2_) chromatography as follows. Each SCX fraction was redissolved in 50 μl of 200 mg/ml 2,5-dihydroxybenzoic acid dissolved in 80% acetonitrile, 2% trifluoroacetic and incubated for 5 min with ∼ 2 mg of TiO2 beads that had been prewashed in the same solution. The beads were then transferred into a ZipTipC18(Millipore) and washed twice with 45 μl of 80% acetonitrile, 2% trifluoroacetic. Bound peptides were eluted with 30 μl of 0.6% ammonia solution, pH 10.5, and the eluted phosphopeptides that absorbed to the underlying C18 material of the ZipTip were eluted with 10 μl of 30% acetonitrile followed by 10 μl of 50% acetonitrile, 0.05% trifluoroacetic acid. The combined eluates were dried, reconstituted in 20 μl of 5% formic acid, vortexed vigorously, and diluted to a final concentration of 1% formic acid with water for LC-MS/MS analysis.

##### Liquid chromatography-mass spectrometry

Liquid chromatography was performed on a fully automated UltiMate Nano LC System (Dionex) fitted with a 1 × 5-mm PepMap C18 trap column and a 75-μm × 15-cm reverse phase PepMap C18 analytical nanocolumn (LC Packings, Dionex). Samples were loaded in 0.1% formic acid (buffer A) and separated using a binary gradient consisting of buffer A and buffer B (90% acetonitrile, 0.08% formic acid). Peptides were eluted with a linear gradient from 5 to 40% buffer B over 130 min. The HPLC system was either coupled to a hybrid quadrupole time-of-flight mass spectrometer (QSTAR XL, Applied Biosystems) or an LTQ-Orbitrap mass spectrometer (Thermo Electron), both equipped with a nanospray ionization source.

##### Software

Chromeleon on the dionex ultimate 3000 and analyst 1.4.1 On the 4000 QTRAP

##### Data analysis

The MSMS data is extracted into a peak list file (it is usually called *.tmp) using a script in Analyst 1.4.1. This peak list file is used by Mascot for database searching.

#### CD spectroscopy

Far UV circular dichroism (CD) spectra were measured with a Jasco J-720 spectropolarimeter using a quartz cuvette with a 1 mm path length in a continuous mode with a bandwidth of 1 nm, response time of 8 s and scan speed of 20 nm˙min^-1^. All measurements were performed at 25°C and each spectrum is an average of four scans. Spectra were recorded in 10 mM Na_2_HPO_4_, 50 mM NaCl (pH = 7.2) with protein concentrations of 50 μM. Background spectra without protein were collected and subtracted from spectra taken in the presence of proteins. Near UV spectra were recorded under the same conditions except that a 1 cm path length and 110 μM protein concentration was used.

#### Modeling the 3D structure of the rat Caskin1 SH3 domain

The 3D structure of the rat Caskin1 SH3 domain was modeled using the public I-TASSER ( http://zhanglab.ccmb.med.umich.edu/I-TASSER) structure prediction server. During the modeling the following template structures were used: NMR solution structure of the human Caskin2 SH3 domain, 2ke9; actin-binding protein SH3 domain from S. cerevisiae, 2k3b; SH3 domain of the human Grb2-like protein 3, 2ew3. The sequence alignment was the following:

LQVRATKDYCNNYDLTSLNVKAGDIITVLEQHPDGRWKGCIHDNRTGNDRVGYFPSSLGEAIV Q8VHK2[284–346]SH3

LKVRALKDFWNLHDPTALNVRAGDVITVLEQHPDGRWKGHIHESQRGTDRIGYFPPGIVEVVS 2ke9A

PWATAEYDYDAA-EDNELTFVENDKIINIEFVDDDWWLGELE----KDGSKGLFPSNYVSLGN 2k3bA

PCCRGLYDFE-PENQGELGFKEGDIITLTNQIDENWYEGMIHG------ESGFFPINYVEVIV 2ew3A

#### Native Gel electrophoresis

Native gel electrophoresis has been used to determine if the in vitro phosphorylation causes dimerization or aggregation of the SH3 domain. Protein samples were run on a 12,5% non-denaturing polyacrylamide gel. The gel was run at 50 V for 2 hrs in a 4°C cold cabinet.

## Abbreviations

Abi: Abl interactor; Caskin: Cask-interacting protein; CD spectroscopy: Circular dichroism spectroscopy; GFP: Green fluorescence protein; IP: Immunoprecipitation; NIK: Nck-interacting kinase; PBS: Phosphate buffered saline; PDGF: Platelet-derived growth factor; PPII: Polyproline II helix; SAM: Sterile α motifs; SH2 domain: Src homology domain 2; SH3 domain: src homology domain 3; SDS-PAGE: Sodium dodecyl sulfate polyacrylamide gel electrophoresis; WASP: Wiskott-Aldrich syndrome protein.

## Competing interests

The authors declare that they have no competing interests.

## Authors’ contributions

SP, AB, and RU performed and designed the experiments. BS and AF purified the SH3 domain, carried out the *in vitro* kinase assay, and measured CD spectra. AF performed the native gel electrophoresis. GB helped to maintain the cells. LB, the corresponding author, supervised the experiments and wrote the manuscript with the help of GB. All authors read and approved the final manuscript.

## Supplementary Material

Additional file 1 Phosphomapping Results of Caskin1 and P-Caskin1. Click here for file
