# Supplementary material for: Complex formation of EphB1/Nck/Caskin1 leads to tyrosine phosphorylation and structural changes of the Caskin1 SH3 domain
Source: Cell Commun Signal. 2012 Nov 27;10:36. doi: 10.1186/1478-811X-10-36 (PMC3549760; doi:10.1186/1478-811X-10-36)
Supplement: Additional file 1 — Phosphomapping Results of Caskin1 and P-Caskin1. [file 1478-811X-10-36-S1.doc]

**Phosphomapping Summary Report**

Sample Name: **Caskin**

Sequence: > NM080690

MGKEQELVQAVKAEDVGTAQRLLQRPRPGKAKLLGSTKKINVNFQDPDGFSALHHAAL

NGNTELISLLLEAQAAVDIKDNKGMRPLHYAAWQGRKEPMKLVLKAGSAVNVPSDEGH

IPLHLAAQHGHYDVSEMLLQHQSNPCMVDNSGKTPLDLACEFGRVGVVQLLLSSNMCA

ALLEPRPGDTTDPNGTSPLHLAAKNGHIDIIRLLLQAGIDINRQTKSGTALHEAALCG

KTEVVRLLLDSGINAQVRNTYSQTALDIVHQFTTSQASKEIKQLLREASAALQVRATK

DYCNNYDLTSLNVKAGDIITVLEQHPDGRWKGCIHDNRTGNDRVGYFPSSLGEAIVKR

AGSRTGSEPSPPQGGGSLGPSAPPEEIWVLRKPFAGGDRSGSLSNVAGGRSTGGHALH

AGAEGVKLLATVLSQKSVSESSPGD**S**PVKPPEGSSGAARSQPPAAHAGQVYGEQPPKK

LESSSASEGKSAEAVSQWLATFQLQLYAPNFTSAGYDLPTISRMTPEDLTAIGVTKPG

HRKKITAEISGLNIPDCLPEHKPANLAVWLSMIGLAQYYKVLVDNGYENIDFITDITW

EDLQEIGITKLGHQKKLMLAVRKLAELQKAEYSKYEGGPLRRKAPQSLEMMAIE**S**PPP

**S**EPAAAECQ**S**PKMTTFQDSELSGELQAALSGPAEAGAAAAEKSSNHLPA**T**PRTTSRQE

SSLSGRARH**M**SS**S**QELLGDGPQGPGSP**M**SR**S**QEYLLDEGPAPG**T**PPKEVRSSRHGHSV

KRA**S**VPPVPGKPRQVLPSGVSHFTPPQTPTKAQPG**S**PQALGGPHGPATAKVKPTPQLL

PPTDRP**M**SPRSLPQSPTHRGFAYVLPQPVEGEAGPPAPGPVPPPVPAAVPTLCLPPEA

DVEPGRPKKRAHSLNRYAASDSEPERDELLVPAAAGPYATVQRRVGRSHSVRAPAGTD

KNVNRSQSFAVRPRKKGPPPPPPKRSSSA**M**ASANLADEP**S**PDVETEDGRLGVRAQRRR

A**S**DLAGSVDTGSAGSVKSIAAMLELSSIGGGGRAIRRPPEGHPTPRPASPDPGRVATV

LASVKHKEAIGPDGEVVNRRR**T**L**S**GPVTGLLATARRGPGEPAEQSHFMEDGTARQRLR

GPAKGEAGVEGPPLARVEASATLKRRIRAKQSQQENVKFILTESDTVKRRPKAKEPDI

GPEPPPPLSVYQNGTATIRRRPASEQAGPPELPPPPPPAEPPPTDL**M**PLPPLPLPDGS

ARKPVKPPV**S**PKPILAQPVSKIQGSPTPASKKVPLPGPG**S**PEVKRAHGTPPPVSPKPP

PPPTAPKPAKALAGLQSSSATPSPVPSPARQPPAALIKPASSPPSQSASPAKPPSPGA

PALQVPTKPPRAAASVVSGPPVASDCASPGDSARQKLEETSACLAAALQAVEEKIRQE

DGQGPRPSSIEEKSTGSILEDIGSMFDDLADQLDAMLEKGNSADIQHSGGRSSLEGPR

FEGLPIPNPLLGLDSTRTGHHHHHH

Mascot (m/z) Pre79 (m/z) Sequence

526.7929 - LLATVLSQK + Phospho (ST)

533.2531 - IQGSPTPASK + Phospho (ST)

551.7567 - SLPQSPTHR + Phospho (ST)

564.2719 - SQSFAVRPR + Phospho (ST)

580.2666 - SSNHLPA**T**PR + Phospho (ST)

628.8150 - VPLPGPG**S**PEVK + Phospho (ST)

653.3016 - SHSVRAPAGTDK + Phospho (ST)

670.8639 - RA**S**VPPVPGKPR + Phospho (ST)

692.8676 - KVPLPGPG**S**PEVK + Phospho (ST)

694.8119 - TTSRQESSLSGR + Phospho (ST)

706.8695 - VPLPGPG**S**PEVKR + Phospho (ST)

718.8805 715.4 TLSGPVTGLLATAR + Phospho (ST)

758.8630 - **T**L**S**GPVTGLLATAR + 2 Phospho (ST)

770.9185 768.4 KVPLPGPG**S**PEVKR + Phospho (ST)

773.8850 - VGYFPSSLGEAIVK + Phospho (ST)

796.9308 - RTL**S**GPVTGLLATAR + Phospho (ST)

801.3587 - A**S**DLAGSVDTGSAGSVK + Phospho (ST)

868.8791 - QEDGQGPRPSSIEEK + Phospho (ST)

860.3672 - QEDGQGPRPSSIEEK + Gln->pyro-Glu (N-term Q); Phospho (ST)

583.6582 - RRTLSGPVTGLLATAR + Phospho (ST)

879.4100 876.4 RA**S**DLAGSVDTGSAGSVK + Phospho (ST)

899.8713 894.5 DYCNNYDLTSLNVK + Phospho (Y)

911.4386 909.5 AQPG**S**PQALGGPHGPATAK + Phospho (ST)

610.3137 - RR**T**L**S**GPVTGLLATAR + 2 Phospho (ST)

919.3927 - RA**S**DLAGSVDTGSAGSVK + 2 Phospho (ST)

939.9335 - **S**QEYLLDEGPAPGTPPK + Phospho (ST)

638.6423 954.5 RRA**S**DLAGSVDTGSAGSVK + Phospho (ST)

661.9918 *-* QVLPSGVSHFTPPQTPTK + Gln->pyro-Glu (N-term Q); Phospho (ST)

1000.9795 - RPPEGHPTPRPASPDPGR + Phospho (ST)

1000.9988 *-* QVLPSGVSHFTPPQTPTK + Phospho (ST)

1002.9505 - GNSADIQHSGGRSSLEGPR + Phospho (ST)

669.3171 - IRQEDGQGPRPSSIEEK + Phospho (ST)

523.3104 1041.5 KPVKPPV**S**PKPILAQPVSK + Phospho (ST)

1055.5500 - VKPTPQLLPPTDRPMSPR + Phospho (ST)

1104.9871 1101.6 AAASVVSGPPVASDCASPGDSAR + Phospho (ST)

1132.0404 1128.6 SQEYLLDEGPAPGTPPKEVR + Phospho (ST)

1133.0062 - SVSESSPGD**S**PVKPPEGSSGAAR + Phospho (ST)

1168.0007 - HMSSSQELLGDGPQGPGSPMSR + Phospho (ST)

781.6832 - SQEYLLDEGPAPG**T**PPKEVR + 2 Phospho (STY)

1175.9937 - H**M**SSSQELLGDGPQGPGSPMSR + Oxidation (M); Phospho (ST)

1183.9947 - H**M**SSSQELLGDGPQGPGSP**M**SR + 2 Oxidation (M); Phospho (ST)

807.3768 - NTYSQTALDIVHQFTTSQASK + Phospho (STY)

1258.5168 - SSSAMASANLADEPSPDVETEDGR + Phospho (ST)

1266.5127 - SSSA**M**ASANLADEP**S**PDVETEDGR + Oxidation (M); Phospho (ST)

860.0467 - ARH**M**SSSQELLGDGPQGPGSPMSR + Oxidation (M); Phospho (ST)

865.3780 - ARH**M**SSSQELLGDGPQGPGSP**M**SR + 2 Oxidation (M); Phospho (ST)

891.3788 - RSSSAMASANLADEPSPDVETEDGR + Phospho (ST)

1391.6619 - TGSEPSPPQGGGSLGPSAPPEEIWVLR + Phospho (ST)

1478.6935 982.5 YAASDSEPERDELLVPAAAGPYATVQR + Phospho (STY)

989.0967 - APQSLEMMAIESPPPSEPAAAECQSPK + 2 Oxidation (M); Phospho (ST)

1012.7871 - YAASDSEPERDELLVPAAAGPYATVQR + 2 Phospho (STY)

1021.1298 - KAPQSLEMMAIESPPPSEPAAAECQ**S**PK + Phospho (ST)

1026.4656 - KAPQSLEMMAIESPPPSEPAAAECQ**S**PK + Oxidation (M); Phospho (ST)

1031.7968 - KAPQSLEMMAIESPPPSEPAAAECQSPK + 2 Oxidation (M); Phospho (ST)

1034.5034 - ASDLAGSVDTGSAGSVKSIAAMLELSSIGGGGR + Phospho (ST)

1051.8420 - AGSRTGSEPSPPQGGGSLGPSAPPEEIWVLR + Phospho (ST)

1053.1189 - KAPQSLEMMAIESPPPSEPAAAECQ**S**PK + Oxidation (M); 2 Phospho (ST)

1058.4511 - KAPQSLEMMAIE**S**PPPSEPAAAECQ**S**PK +2 oxidation (M); 2 Phospho (ST)

1073.5415 - LLATVLSQKSVSESSPGDSPVKPPEGSSGAAR + Phospho (ST)

1083.8300 - RKAPQSLEMMAIESPPPSEPAAAECQ**S**PK + 2 Oxidation (M); Phospho (ST)

971.7717 - QPPAALIKPASSPPSQSASPAKPPSPGAPALQVPTKPPR +

Gln->pyro-Glu (N-term Q); Phospho (ST)

976.0317 - QPPAALIKPASSPPSQSASPAKPPSPGAPALQVPTKPPR + Phospho (ST)

1322.0125 - QPPAALIKPASSPPSQSASPAKPPSPGAPALQVPTKPPR +

Gln->pyro-Glu (N-term Q); 2 Phospho (ST)

1327.6865 - QPPAALIKPASSPPSQSASPAKPPSPGAPALQVPTKPPR + 2 Phospho (ST)

1377.6201 - HMSSSQELLGDGPQGPGSP**M**SRSQEYLLDEGPAPGTPPK +

Oxidation (M); Phospho (STY)

1037.4700 - HMSSSQELLGDGPQGPGSPMSRSQEYLLDEGPAPGTPPK +

2 Oxidation (M); Phospho (ST)

1087.8053 - RRPASEQAGPPELPPPPPPAEPPPTDL**M**PLPPLPLPDGSAR.K

+ Oxidation (M); Phospho (ST)

532.2772 1060.5 VKPTPQLLPPTDRP**M**SPR.S + Oxidation (M); Phospho (ST)

Sample Name: **P-Caskin**

Sequence: > NM080690

MGKEQELVQAVKAEDVGTAQRLLQRPRPGKAKLLGSTKKINVNFQDPDGFSALHHAAL

NGNTELISLLLEAQAAVDIKDNKGMRPLHYAAWQGRKEPMKLVLKAGSAVNVPSDEGH

IPLHLAAQHGHYDVSEMLLQHQSNPCMVDNSGKTPLDLACEFGRVGVVQLLLSSNMCA

ALLEPRPGDTTDPNGTSPLHLAAKNGHIDIIRLLLQAGIDINRQTKSGTALHEAALCG

KTEVVRLLLDSGINAQVRNTYSQTALDIVHQFTTSQASKEIKQLLREASAALQVRATK

D**Y**CNN**Y**DLTSLNVKAGDIITVLEQHPDGRWKGCIHDNRTGNDRVG**Y**FPSSLGEAIVKR

AGSRTGSEPSPPQGGGSLGPSAPPEEIWVLRKPFAGGDRSGSLSNVAGGRSTGGHALH

AGAEGVKLLATVLSQKSVSESSPGDSPVKPPEGSSGAARSQPPAAHAGQVYGEQPPKK

LESSSASEGKSAEAVSQWLATFQLQLYAPNFTSAGYDLPTISRMTPEDLTAIGVTKPG

HRKKITAEISGLNIPDCLPEHKPANLAVWLSMIGLAQYYKVLVDNGYENIDFITDITW

EDLQEIGITKLGHQKKLMLAVRKLAELQKAEYSKYEGGPLRRKAPQSLE**MM**AIESPPP

SEPAAAECQ**S**PKMTTFQDSELSGELQAALSGPAEAGAAAAEKSSNHLPA**T**PRTTSRQE

SSLSGRARH**M**SSSQELLGDGPQGPGSP**M**SRSQEYLLDEGPAPG**T**PPKEVRSSRHGHSV

KRA**S**VPPVPGKPRQVLPSGVSHFTPPQTPTKAQPG**S**PQALGGPHGPATAKVKPTPQLL

PPTDRPMSPRSLPQSPTHRGFAYVLPQPVEGEAGPPAPGPVPPPVPAAVPTLCLPPEA

DVEPGRPKKRAHSLNRYAASDSEPERDELLVPAAAGPYATVQRRVGRSHSVRAPAGTD

KNVNRSQSFAVRPRKKGPPPPPPKRSSSA**M**ASANLADEPSPDVETEDGRLGVRAQRRR

A**S**DLAGSVDTGSAG**S**VKSIAAMLELSSIGGGGRAIRRPPEGHPTPRPA**S**PDPGRVATV

LASVKHKEAIGPDGEVVNRRR**T**L**S**GPVTGLLATARRGPGEPAEQSHFMEDGTARQRLR

GPAKGEAGVEGPPLARVEASATLKRRIRAKQSQQENVKFILTESDTVKRRPKAKEPDI

GPEPPPPLSVYQNGTATIRRRPASEQAGPPELPPPPPPAEPPPTDLMPLPPLPLPDGS

ARKPVKPPV**S**PKPILAQPVSKIQGSPTPASKKVPLPGPG**S**PEVKRAHGTPPPVSPKPP

PPPTAPKPAKALAGLQSSSATPSPVPSPARQPPAALIKPASSPPSQSASPAKPPSPGA

PALQVPTKPPRAAASVVSGPPVASDCA**S**PGDSARQKLEETSACLAAALQAVEEKIRQE

DGQGPRPSSIEEKSTGSILEDIGSMFDDLADQLDAMLEKGNSADIQHSGGRSSLEGPR

FEGLPIPNPLLGLDSTRTGHHHHHH

Mascot (m/z) Pre79 (m/z) Sequence

526.7927 - LLATVLSQK + Phospho (ST)

533.2529 - IQGSPTPASK + Phospho (ST)

542.7434 540.3 SGSLSNVAGGR + Phospho (ST)

551.7564 - SLPQSPTHR + Phospho (ST)

564.2719 563.3 SQSFAVRPR + Phospho (ST)

580.2669 - SSNHLPA**T**PR + Phospho (ST)

628.8184 - VPLPGPG**S**PEVK + Phospho (ST)

653.3013 - SHSVRAPAGTDK + Phospho (ST)

670.8639 - RA**S**VPPVPGKPR + Phospho (ST)

462.2464 689.3 KVPLPGPG**S**PEVK + Phospho (ST)

694.8121 - TTSRQESSLSGR + Phospho (ST)

706.8685 - VPLPGPG**S**PEVKR + Phospho (ST)

718.8809 716.3 TLSGPVTGLLATAR + Phospho (ST)

725.3227 - AEYSKYEGGPLR + Phospho (STY)

758.8629 756.4 **T**L**S**GPVTGLLATAR + 2 Phospho (ST)

770.9165 768.4 KVPLPGPG**S**PEVKR + Phospho (ST)

773.8832 - VG**Y**FPSSLGEAIVK + Phospho (Y)

796.9308 - R**T**LSGPVTGLLATAR + Phospho (ST)

801.3564 - ASDLAGSVDTGSAGSVK + Phospho (ST)

568.2911 - VGYFPSSLGEAIVKR + Phospho (STY)

868.8792 - QEDGQGPRPSSIEEK + Phospho (ST)

583.6576 ***-*** RR**T**LSGPVTGLLATAR + Phospho (ST)

879.4092 876.5 RA**S**DLAGSVDTGSAGSVK + Phospho (ST)

899.8714 - D**Y**CNNYDLTSLNVK + Phospho (Y)

911.4383 909.5 AQPG**S**PQALGGPHGPATAK + Phospho (ST)

610.3124 ***-***  RR**T**L**S**GPVTGLLATAR + 2 Phospho (ST)

919.3926 - RA**S**DLAGSVDTGSAG**S**VK + 2 Phospho (ST)

939.9301 - SQEYLLDEGPAPGTPPK + Phospho (STY)

992.4835 - QVLPSGVSHFTPPQTPTK + Gln->pyro-Glu (N-term Q); Phospho (ST)

1000.9784 - RPPEGHPTPRPA**S**PDPGR + Phospho (ST)

1000.9952 - QVLPSGVSHFTPPQTPTK + Phospho (ST)

1002.9503 - GNSADIQHSGGRSSLEGPR + Phospho (ST)

669.31664 - IRQEDGQGPRPSSIEEK + Phospho (ST)

1045.6140 1043.5 KPVKPPV**S**PKPILAQPVSK + Phospho (ST)

1049.9633 - ATKDYCNN**Y**DLTSLNVK + Phospho (Y)

1055.5473 - VKPTPQLLPPTDRPMSPR + Phospho (ST)

1089.9457 - ATKDYCNN**Y**DLTSLNVK + 2 Phospho (STY)

1104.9870 - AAASVVSGPPVASDCA**S**PGDSAR + Phospho (ST)

1132.0379 1128.6 SQEYLLDEGPAPG**T**PPKEVR + Phospho (ST)

1133.0059 - SVSESSPGDSPVKPPEGSSGAAR + Phospho (ST)

1167.9977 - HMSSSQELLGDGPQGPGSPMSR + Phospho (ST)

1175.9937 - H**M**SSSQELLGDGPQGPGSPMSR + Oxidation (M); Phospho (ST)

1183.9931 - H**M**SSSQELLGDGPQGPGSP**M**SR + 2 Oxidation (M); Phospho (ST)

1210.5643 - NTYSQTALDIVHQFTTSQASK + Phospho (STY)

810.9867 - HMSSSQELLGDGPQGPGSPM**S**R + Oxidation (M); 2 Phospho (ST)

1258.5120 - SSSAMASANLADEPSPDVETEDGR + Phospho (ST)

1266.5115 - SSSA**M**ASANLADEPSPDVETEDGR + Oxidation (M); Phospho (ST)

860.0456 - ARH**M**SSSQELLGDGPQGPGSPMSR + Oxidation (M); Phospho (ST)

865.3774 - ARH**M**SSSQELLGDGPQGPGSP**M**SR + 2 Oxidation (M); Phospho (ST)

891.3785 - RSSSAMASANLADEPSPDVETEDGR + Phospho (ST)

909.7852 - AKEPDIGPEPPPPLSVYQNGTATIR + Phospho (STY)

1391.6599 - TGSEPSPPQGGGSLGPSAPPEEIWVLR + Phospho (ST)

738.0750 - APQSLEMMAIESPPPSEPAAAECQ**S**PK + Oxidation (M); Phospho (ST)

1478.6952 983.54 YAASDSEPERDELLVPAAAGPYATVQR + Phospho (STY)

742.0744 - APQSLEMMAIESPPPSEPAAAECQ**S**PK + 2 Oxidation (M); Phospho (ST)

1012.7879 - YAASDSEPERDELLVPAAAGPYATVQR + 2 Phospho (STY)

1015.7519 - APQSLEMMAIESPPPSEPAAAECQ**S**PK + 2 Oxidation (M); 2 Phospho (ST)

1021.1306 - KAPQSLEMMAIESPPPSEPAAAECQSPK + Phospho (ST)

770.0996 - KAPQSLEMMAIESPPPSEPAAAECQ**S**PK + Oxidation (M); Phospho (ST)

1031.7955 - KAPQSLEMMAIESPPPSEPAAAECQSPK + 2 Oxidation (M); Phospho (ST)

1039.4417 - YAASDSEPERDELLVPAAAGPYATVQR + 3 Phospho (STY)

1051.8423 - AGSRTGSEPSPPQGGGSLGPSAPPEEIWVLR + Phospho (ST)

1053.1197 - KAPQSLEMMAIESPPPSEPAAAECQ**S**PK + Oxidation (M); 2 Phospho (ST)

1058.4511 - KAPQSLEMMAIESPPPSEPAAAECQ**S**PK +2 oxidation (M); 2 Phospho (ST)

1073.5412 - LLATVLSQKSVSESSPGDSPVKPPEGSSGAAR + Phospho (ST)

809.1219 - RKAPQSLEMMAIESPPPSEPAAAECQ**S**PK + Oxidation (M); Phospho (ST)

1295.3587 - QPPAALIKPASSPPSQSASPAKPPSPGAPALQVPTKPPR +

Gln->pyro-Glu (N-term Q); Phospho (ST)

1322.0125 - QPPAALIKPASSPPSQSASPAKPPSPGAPALQVPTKPPR +

Gln->pyro-Glu (N-term Q); 2 Phospho (ST)

1377.6214 - HMSSSQELLGDGPQGPGSPMSRSQEYLLDEGPAPGTPPK +

Oxidation (M); Phospho (STY)

1382.9550 - HMSSSQELLGDGPQGPGSPMSRSQEYLLDEGPAPGTPPK +

2 Oxidation (M); Phospho (ST)

957.4589 954.5 RRA**S**DLAGSVDTGSAGSVK + Phospho (ST)

709.3667 1059.58 VKPTPQLLPPTDRPMSPR.S + Oxidation (M); Phospho (ST)

Abbreviations:

M- Indicated oxidized methionine

**S T Y**- Indicated phosphorylation site assigned

S T Y - Indicated ambiguous assignment-could be any of residues highlighted
